# High density genotype storage for plant breeding in the Chado schema of Breedbase

**DOI:** 10.1371/journal.pone.0240059

**Published:** 2020-11-11

**Authors:** Nicolas Morales, Guillaume J. Bauchet, Titima Tantikanjana, Adrian F. Powell, Bryan J. Ellerbrock, Isaak Y. Tecle, Lukas A. Mueller

**Affiliations:** 1 Plant Breeding and Genetics, Cornell University, Ithaca, NY, United States of America; 2 Boyce Thompson Institute, Ithaca, NY, United States of America; Mayo Clinic Arizona, UNITED STATES

## Abstract

Modern breeding programs routinely use genome-wide information for selecting individuals to advance. The large volumes of genotypic information required present a challenge for data storage and query efficiency. Major use cases require genotyping data to be linked with trait phenotyping data. In contrast to phenotyping data that are often stored in relational database schemas, next-generation genotyping data are traditionally stored in non-relational storage systems due to their extremely large scope. This study presents a novel data model implemented in Breedbase (https://breedbase.org/) for uniting relational phenotyping data and non-relational genotyping data within the open-source PostgreSQL database engine. Breedbase is an open-source, web-database designed to manage all of a breeder’s informatics needs: management of field experiments, phenotypic and genotypic data collection and storage, and statistical analyses. The genotyping data is stored in a PostgreSQL data-type known as binary JavaScript Object Notation (JSONb), where the JSON structures closely follow the Variant Call Format (VCF) data model. The Breedbase genotyping data model can handle different ploidy levels, structural variants, and any genotype encoded in VCF. JSONb is both compressed and indexed, resulting in a space and time efficient system. Furthermore, file caching maximizes data retrieval performance. Integration of all breeding data within the Chado database schema retains referential integrity that may be lost when genotyping and phenotyping data are stored in separate systems. Benchmarking demonstrates that the system is fast enough for computation of a genomic relationship matrix (GRM) and genome wide association study (GWAS) for datasets involving 1,325 diploid *Zea mays*, 314 triploid *Musa acuminata*, and 924 diploid *Manihot esculenta* samples genotyped with 955,690, 142,119, and 287,952 genotype-by-sequencing (GBS) markers, respectively.

## Introduction

Routine genotyping is now possible with the advent of low-cost, high-throughput genotyping platforms, giving rise to enormous amounts of data but presenting challenges for data management and queriability [1]. Plant breeding programs routinely genotype for 1) quality control and validation of experimental designs [2, 3], 2) trait discovery through genome-wide association studies (GWAS), and 3) prediction of phenotypic performance via marker-assisted selection and genomic selection (GS) [4–7]. To serve these three scenarios effectively and efficiently, it is critical to store germplasm, pedigrees, experimental designs, and phenotypic and genotypic information under a unified architecture. These services can either be implemented within a single database or provided by independent applications interconnected via application programming interfaces (APIs) such as, the publicly specified Plant Breeding API (BrAPI) [8]. Here, we report the implementation of all the aforementioned services under a single web-application connecting to a single database backend in the Breedbase system.

Breedbase is an open-source web-based database currently used by dozens of plant breeding communities, including https://cassavabase.org and https://solgenomics.net [9–11]. The codebase and application deployment are available from https://github.com/solgenomics/sgn and https://github.com/solgenomics/breedbase_dockerfile, respectively.

For the backend database, Breedbase runs on PostgreSQL, an open-source relational database engine [12]. Breedbase uses the Chado schema, supplemented with custom schema extensions for handling user accounts and other metadata. Chado was initially developed at Flybase and is now an important part of the Generic Model Organism Database (GMOD) suite of tools; Chado is designed to be modular and ontology driven [13, 14]. Ontology driven schemas use controlled vocabularies to map data spaces; they can be specific to an application or they can be open and shared across many databases. One successful example of open-access controlled vocabularies is the Crop Ontology project, which enables a common vocabulary for evaluating crops [15]. Adhering to an ontology driven philosophy enables Chado to be highly flexible in handling the multitude of database implementations it currently serves. At the time of writing, there are 220 genomic databases that use some or all the modules in Chado [16]. Critical to Breedbase and the work described here, is the Natural Diversity (ND) module in Chado, which is designed to store data relating to any experiment involving phenotyping and/or genotyping [17].

Chado has traditionally been a purely relational schema. Non-relational or NoSQL databases such as MongoDB, have recently become very popular, particularly for high density data-types, such as genotyping data [18]. An advantage of non-relational databases is that relationships do not have to be explicitly defined in a schema. Instead, data can be stored via nested objects composed of heterogeneous keys and values, allowing for flexibility in the data structure and model; often non-relational data is structured using JavaScript Object Notation (JSON). This is ideal for large, rapidly changing, or unstructured data. However, a disadvantage of non-relational databases is that they are optimized for data retrieval, not for updating data or the relationships between data. Given the broad community interest in querying non-relational JSON structures concurrently with traditional relational data, the SQL specification has evolved to support JSON storage and querying [19]. To accommodate the new standards, PostgreSQL, which Breedbase uses, has added support for JSON and JSONb column data-types. JSONb is a binary formatted JSON field, allowing for compressed data sizes and faster queries in some scenarios.

Breeding methods such as GWAS and GS depend on large genotypic data and metadata, generally stored in a standardized Variant Call Format (VCF) structure. VCF is a generic file format for storing sequence variation, such as SNPs, indels, and structural variants, along with annotations [20]. This format enables interoperability between researchers and between software programs, as well as simple file generation and compact data representation for large numbers of samples and markers. The JSON genotype storage model presented here closely follows the VCF specification and can handle any kind of variant encoded in VCF, such as different ploidy levels, multiple alleles, insertions or deletions (indels), and structural variants. The preferred format for uploading and downloading genotypic data in Breedbase is VCF.

## Materials and methods

### I. Chado schema modifications

**Fig 1:**
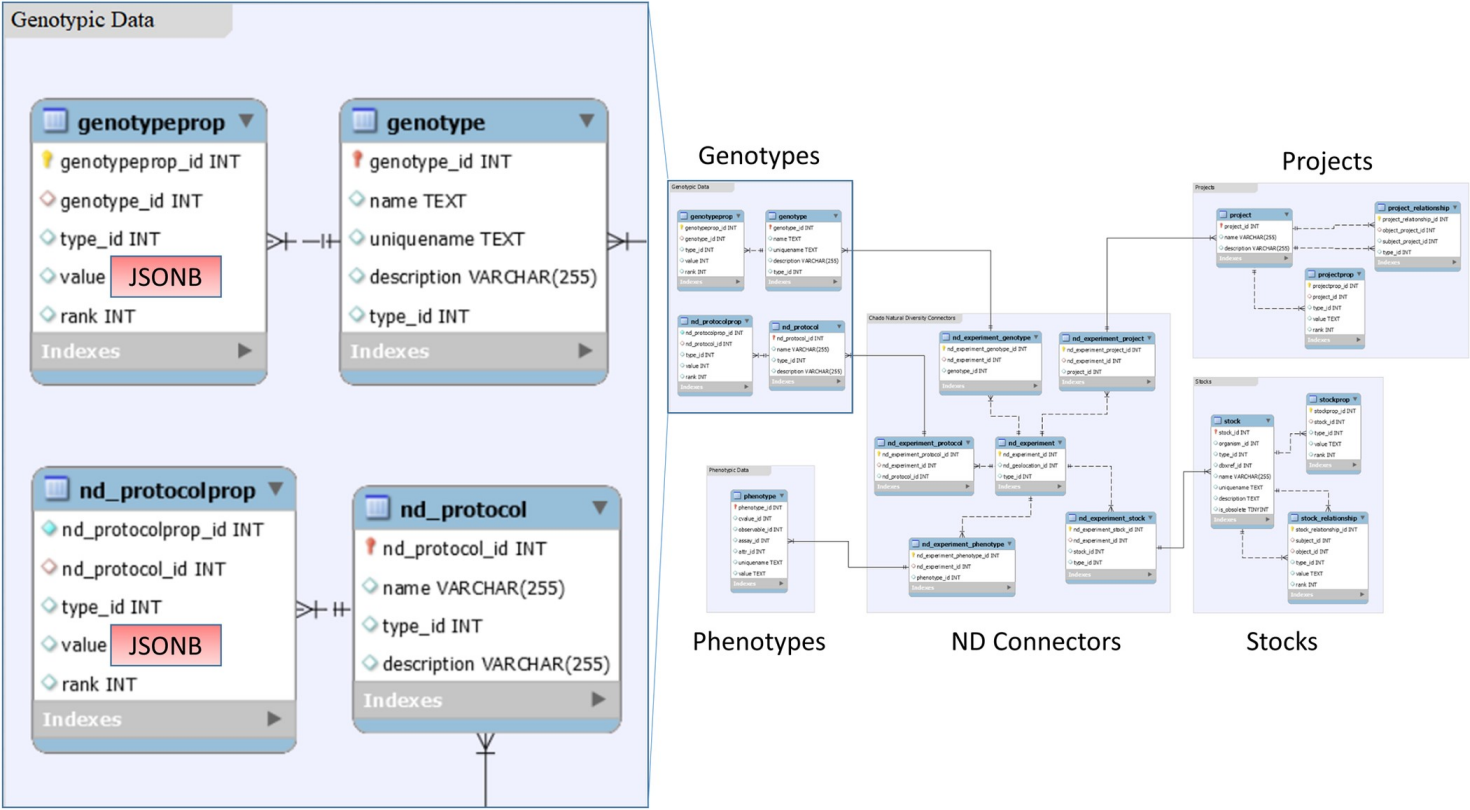
Core Chado database schema relied upon by Breedbase. The modifications for non-relationally storing genotyping data within the Chado relational schema are highlighted in red [17].

The Natural Diversity (ND) module [17] in the Chado schema provides the foundation for the database schema used in the following work. The ND module allows for storage and querying over many projects, and the evaluation of many stocks, genotyping experiments, and phenotyping experiments. Only three modifications to the ND schema are required to accommodate the new non-relational genotyping data storage model: 1) the *value* field in the *genotypeprop* table and 2) the *value* field in the *nd_protocolprop* table are both converted to the JSON (JSONb in PostgreSQL) column type instead of text. Fig 1 shows the core of the schema with the modifications highlighted in red. Additionally, 3) a Generalized Inverted Index (GIN) is applied to the JSONb column in the *genotypeprop* table, allowing for faster queries of keys within the JSON structures.

Genotype data in this schema are linked to a ‘genotyping protocol’; a ‘genotyping protocol’ is given a name, description, and controlled vocabulary type as an entry in the *nd_protocol* table. The ‘genotyping protocol’ aggregates all meta-information provided in a VCF file header, including variant calling software, genotypes calling format (i.e. allele depth, depth of coverage, quality), the reference genome used for alignment, and marker-related information (i.e. applied filters and information fields). It stores the aforementioned information and all meta-data lines from the VCF file header in a JSON formatted *value* entry of the *nd_protocolprop* table linked to *nd_protocol*. The ‘genotyping protocol’ is central to grouping and delineating marker information in the database. The JSON data structure storage in the *value* field of the *nd_protocolprop* table is described in more detail in the ‘Genotype Storage JSON Structures’ section below. A ‘genotyping project’ is defined as a group of ‘genotyping protocols’, hierarchically; this organizational structure is useful for large breeding programs where multiple genotyping events are occurring. The ‘genotyping project’ is stored as an entry in the *project* table.

The samples genotyped are stored as entries in the *stock* table; in Breedbase, the *stock* entries that can be genotyped have controlled vocabulary terms of either ‘tissue_sample’, ‘plot’, ‘plant’, or ‘accession’. The genotype for a sample under a specific ‘genotyping protocol’ is stored as an entry in the *genotype* table with a link to the *genotypeprop* table; the JSON formatted *value* field in the *genotypeprop* table represents the sample’s complete ‘callset’ under a specific ‘genotyping protocol’. A ‘callset’ represents all genotypes for all markers in the ‘genotyping protocol’ for a single sample as defined in BrAPI [8]. More information on the JSON data structure stored in the *value* field of the *genotypeprop* table is given in the ‘Genotype Storage JSON Structures’ section below. Phenotypes for samples are linked using the *phenotype* table, where the variable being measured is a controlled vocabulary type linked using the *cvalue_id* field and the phenotypic result is stored as a string in the *value* field. The *nd_experiment* table connects the *project*, *nd_protocol*, *stock*, *phenotype*, and *genotype* tables under a controlled vocabulary type, enabling querying across all entities involved.

### II. Genotype storage JSON structures

The JSON *value* fields stored in the *genotypeprop* and *nd_protocolprop* tables take their forms from the VCF data model. Intuitively, the first nine columns of information in the VCF are aggregated into a single JSON object and stored as an entity in the *nd_protocolprop* table, while a new JSON object is aggregated for each of the sample columns (columns 10 to the maximum number of columns in the VCF) and stored as entries in the *genotypeprop* table. Tables 1 and 2 describe the structure of the *value* field JSON objects in the *nd_protocolprop* and *genotypeprop* tables, respectively, for a hypothetical ‘genotyping protocol’ involving two markers named ‘S2_20032’ and ‘S2_20033’.

**Table 1:** Descriptions of the JSON objects stored in the *nd_protocolprop* table.

Controlled Vocabulary Entry	Top-Level Key	Value Data-Type	Description	Example Value
‘vcf_map_details’	reference_genome_name	String	The name of the reference genome against which genotypic variants were called.	‘Mesculenta_511_v7’
‘vcf_map_details’	species_name	String	The name of the species which was genotyped.	‘Manihot esculenta’
‘vcf_map_details’	sample_observation_unit_type_name	String Enum	The controlled vocabulary type of the sample which was genotyped. Can be either ‘tissue_sample’, ‘plant’, ‘plot’, or ‘accession’ in the Breedbase database.	‘tissue_sample’
‘vcf_map_details’	header_information_lines	Array of Strings	An array containing information about the genotyping protocol. Intended for the information lines at the top of the VCF beginning with “##”.	[ "##fileformat = VCFv4.0", "##Tassel = <ID = GenotypeTable, Version = 5>", "##FORMAT = <ID = GT, Number = 1, Type = String, Description = 'Genotype'>" ]
‘vcf_map_details’	marker_names	Array of Strings	An array containing all marker names used in the genotyping protocol.	[ "S2_20032", "S2_20033" ]
‘vcf_map_details_markers’	None	Object	An object where the top-level keys are the marker names and the respective values are marker information objects (MIOs). This object contains all markers involved in the genotyping protocol.	{ "S2_20032" : { "name" : "S2_20032", "chrom" : "2", "pos" : "20032", "alt" : "G,T", "ref" : "C", "qual" : "99", "filter" : "PASS", "info" : "AR2 = 0.29;DR2 = 0.342;AF = 0.375", "format" : "GT:AD:DP:GQ:DS:PL:NT" }, "S2_20033" : { "name" : "S2_20033", "chrom" : "2", "pos" : "20033", "alt" : "G", "ref" : "C", "qual" : "99", "filter" : "PASS", "info" : "AR2 = 0.29;DR2 = 0.342;AF = 0.375", "format" : "GT:AD:DP:GQ:DS:PL:NT" } }
‘vcf_map_details_markers_array’	None	Array of Objects	An array containing marker information objects (MIOs). This array contains all markers used in the genotyping protocol.	[ { "name" : "S2_20032", "chrom" : "2", "pos" : "20032", "alt" : "G", "ref" : "C", "qual" : "99", "filter" : "PASS", "info" : "AR2 = 0.29;DR2 = 0.342;AF = 0.375", "format" : "GT:AD:DP:GQ:DS:PL:NT" }, { "name" : "S2_20033", "chrom" : "2", "pos" : "20033", "alt" : "G", "ref" : "C", "qual" : "99", "filter" : "PASS", "info" : "AR2 = 0.29;DR2 = 0.342;AF = 0.375", "format" : "GT:AD:DP:GQ:DS:PL:NT" } ]

An example genotyping protocol containing two markers named “S2_20032” and “S2_20033” is given.

**Table 2:** Description of JSON object stored in the *genotypeprop* table.

Top-Level Key	Value Data-Type	Description	Example Value
$marker_name (Variable. To follow the example genotyping protocol in Table 1, $marker_name would be “S2_20032” or “S2_20033”.)	Object	The top-level keys are the marker names. Each value is an object containing all genotype score information in accordance with the VCF data model, and the “NT” key. The “NT” key is generated by Breedbase and is important for allelic interpretation of the genotype. The “DS” key represents the dosage genotype (value of ‘0’, ‘1’, ‘2’, etc., or ‘NA’); it is generated by Breedbase if it is not provided during upload.	{ "GT" : "0/0", “NT” : “A,A” "AD" : "9,0", "DP" : "9", "GQ" : "99", "DS": "0", "PL" : "0,27,255" }

The *nd_protocolprop* table stores three entries to describe a genotyping protocol, as described in Table 1. The first entry is stored using the controlled vocabulary term ‘vcf_map_details’ and the *value* field JSON object contains the following top-level keys: ‘reference_genome_name’, ‘species_name’, ‘header_information_lines’, ‘sample_observation_unit_type_name’, and ‘marker_names’. Each of these keys gives information about the genotyping protocol and how the genotyping data was generated. Particularly, the ‘header_information_lines’ key is to store an array of strings for the meta-information header lines found at the top of a VCF file, indicated with ‘##’. The ‘reference_genome_name’, ‘species_name’, and ‘sample_observation_unit_type_name’ keys map to string values for the name of the reference genome used for alignment, the name of the species, and the controlled vocabulary type of the observation unit sampled for genotyping (either ‘tissue_sample’, ‘plant’, ‘plot’, or ‘accession’ in Breedbase). The ‘marker_names’ key stores a list of all marker names used in the protocol.

The second entry in the *nd_protocolprop* table is stored using the controlled vocabulary term ‘vcf_map_details_markers’. In this entry, the *value* field JSON object is a key value mapping of marker names to marker information objects (MIOs); each MIO has the following keys: ‘chrom’, ‘pos’, ‘name’, ‘ref’, ‘alt’, ‘qual’, ‘filter’, ‘info’, and ‘format’, signifying values for, chromosome number, base pair position, marker name or unique identifier, reference allele, comma separated alternate alleles, marker quality score, filter status, additional information, and genotype score format, respectively. This terminology is taken directly from the VCF specification and the meanings and data formats are identical. The third entry in the *nd_protocolprop* table is stored under the controlled vocabulary term ‘vcf_map_details_markers_array’. This entry contains an array of MIOs. Note that the previous entry was an object of MIOs and represents redundant information; however, these two data representations allow for flexible and performant construction of JSON SQL queries in PostgreSQL.

The *value* field JSON object in the *genotypeprop* table is composed of dynamic top-level keys; the top-level keys are all the marker names tested in the ‘genotyping protocol’ and are the same marker names stored in the *value* field JSON object of the *nd_protocolprop* table described in the previous paragraph. As described in Table 2, the subsequent object under these top-level keys contains the genotype score information, with keys coming dynamically from the ‘format’ field of the uploaded genotype file; uploading a VCF file allows for storage of all VCF defined genotype attributes e.g. ‘GT’, ‘AD’, ‘DP’, ‘GQ’, ‘PL’, and ‘GL’. Alternatively, uploading a tab-delimited allele matrix file, allows for storage of only ‘GT’ in the *genotypeprop* JSON object. In all cases for every genotype, the uploaded genotype data files must minimally contain the marker name, the reference and alternate alleles, and the genotype encoded in VCF ‘GT’ form. Additionally in all cases of genotype upload, Breedbase generates and stores two new keys named ‘NT’ and ‘DS’. The ‘NT’ key contains the nucleotides for the given polymorphism (e.g. ‘A’, ‘T’, ‘C’, or ‘G’), separated by a comma; the order of the nucleotides is the same as in the ‘GT’ key and this is true for both unphased data (the GT key contains the “/” separator) and phased data (the GT key contains the “|”separator). The ‘NT’ key is important in the Breedbase system to provide allelic context to the genotype and enables simple querying. The ‘DS’ key represents the dosage genotype and is a value of either ‘0’, ‘1’, ‘2’, etc., or ‘NA’. If the ‘DS’ key is not provided in the VCF it is calculated by Breedbase as a sum of the reference calls in the ‘GT’ key; for instance, where ‘GT’ = '1/1' then ‘DS’ = ‘0’, where ‘GT’ = '0/0’ then ‘DS’ = ‘2’, where ‘GT’ = '0/1' or '1/0' then ‘DS’ = ‘1’, and where ‘GT’ = './.' then ‘DS’ = 'NA'. The ‘DS’ key is important for easily querying dosage genotype results and for subsequent downstream analysis, such as imputation, GWAS or GS. In this way, all genotype information provided in the uploaded genotyping file is stored, with VCF files being the preferred format for uploading genotypic data into Breedbase.

An important difference between a VCF file and the data structure in the database is that VCF files are ‘marker first’ (markers define the rows and the samples define the columns) whereas this database implementation stores the data ‘sample first’ (the database rows define samples and the ‘columns’ in the JSONb data structure define the markers) [21]. The data in the database is therefore essentially the transpose of the VCF file and accordingly has to be transposed when loaded and when downloaded into certain formats, including VCF. Transposition is both memory and time intensive, with a complexity of O(n^2^). While it would be easy to store both ‘marker first’ and ‘sample first’ matrix representations in the database, we have opted not to do this because it leads to complex operations when data have to be added or removed.

### III. Caching of results

Application performance is critical for breeding programs to effectively incorporate genotypic information in decision making. Provided that genotyping protocols do not change after being uploaded into Breedbase, file caching is used to maximize genotypic query performance in the Breedbase system and to minimize system memory requirements. When a user issues a genotype query to the Breedbase system the following actions are executed: (1) the query parameters are encoded into an MD5 hash string (2) the file cache determines whether this string represents a query that has not been served by the system before (3) if the query has been served before then the results will simply be returned to the user from the file cache (4) if the query has not been served before, then the results will be fetched from the PostgreSQL database, written to the file cache, and then returned to the user. The file cache minimizes system memory requirements by iteratively writing results from the PostgreSQL database to the cached file line-by-line; similarly, results can be efficiently read line-by-line.

The file cache is implemented using the Perl Cache::File module. To meet the requirements of different analyses applications, three formats can be retrieved from the file cache system currently: VCF, dosage matrix, and internal JSON. As is discussed in the “Packaged Queries” section below, simple entry-points for retrieving results in any of the three formats from the file cache are provided. The VCF and dosage matrix formats are largely for user-facing actions for serving genotype results directly as files, whereas the internal JSON format is largely for operations and tools within Breedbase. In the dosage matrix format, the first column lists all the genotyped markers, each subsequent column is for a genotyped sample, and the genotypes are dosage values (e.g. ‘0’, ‘1’, ‘2’, etc., or ‘NA’). The internal JSON format follows the JSON representations previously described; however, it also returns experimental metadata concerning the genotyped samples.

## Results

### I. Example SQL queries

Using PostgreSQL’s JSON and JSONb query functions, precise queries spanning phenotypes and genotypes can be constructed; however, directly constructing SQL queries is not recommended because it does not take advantage of the file caching system within Breedbase. Furthermore, constructing SQL queries directly may not retrieve all the possible metadata that is available within the Breedbase database. The queries here are for demonstration purposes only; in practice, the examples demonstrated in the “Packaged Queries” section below should be used as templates in production settings.

#### SQL Example I

To construct a query with the following criteria: (1) stocks genotyped for a marker named either ‘S10_0880’ or ‘S11_0112’, and (2) stocks have been phenotyped for a trait called ‘plant height in cm’ in a field phenotyping experiment called ‘2019_CA_MT’, an SQL query can be written as:

SELECT stock.uniquename FROM stock
JOIN nd_experiment_stock USING (stock_id)
JOIN nd_experiment_phenotype USING (nd_experiment_id)
JOIN nd_experiment_project USING (nd_experiment_id)
JOIN nd_experiment_protocol USING (nd_experiment_id)
JOIN phenotype USING (phenotype_id)
JOIN project USING (project_id)
JOIN nd_protocolprop USING (nd_protocol_id)
JOIN cvterm ON (phenotype.cvalue_id = cvterm.cvterm_id)
WHERE cvterm.name = ‘plant height in cm’
AND project.name = ‘2019_CA_MT’
AND nd_protocolprop.value->‘markers’?| array[‘S10_0880’, ‘S11_0112’];

#### SQL Example II

To construct a query with the following criteria: (1) all triploid stocks were genotyped for a marker named ‘S8_0880’ in a genotyping protocol named ‘2019_GT_MAP’ with genotyping depth of coverage (‘DP’) greater than 10 and genotyping quality (‘GQ’) greater than 90 and (2) have the ‘T’ allele on all chromosomes in an unphased call where the ‘T’ allele is the reference allele, and (3) were phenotyped for a trait called ‘plant height in cm’ at a value greater than 5, an SQL query could be written as:

SELECT stock.uniquename FROM stock
JOIN nd_experiment_stock USING (stock_id)
JOIN nd_experiment_phenotype USING (nd_experiment_id)
JOIN nd_experiment_protocol USING (nd_experiment_id)
JOIN nd_experiment_genotype USING (nd_experiment_id)
JOIN phenotype USING (phenotype_id)
JOIN nd_protocol USING (nd_protocol_id)
JOIN nd_protocolprop USING (nd_protocol_id)
JOIN genotypeprop USING (genotype_id)
JOIN cvterm ON (phenotype.cvalue_id = cvterm.cvterm_id)
WHERE cvterm.name = ‘plant height in cm’
AND phenotype.value::int > 5
AND nd_protocol.name = ‘2019_GT_MAP’
AND nd_protocolprop.value->‘markers’->‘S10_0880’->‘DP’::int > 10
AND nd_protocolprop.value->‘markers’->‘S10_0880’->‘GQ’::int > 90
AND genotypeprop.value->‘S8_0880’->‘NT’ = ‘T,T,T’
AND genotypeprop.value->‘S8_0880’->‘GT’ = ‘0/0/0’

### II. Packaged queries

Perl Moose objects named CXGN::Genotype::Search, CXGN::Genotype::GRM, and CXGN::Genotype::GWAS are available in Breedbase to facilitate query and analyses construction, and to provide an interface to the file cache system.

#### A. CXGN::Genotype::Search

The CXGN::Genotype::Search object allows genotypes to be queried for specific accessions, tissue samples, field trials, genotyping protocols, markers, chromosomes, and base pair positions, using the ‘accession_list’, ‘tissue_sample_list’, ‘trial_list’, ‘protocol_id_list’, ‘marker_name_list’, ‘chromosome_list’, and ‘start_position’ and ‘end_position’ parameters, respectively. Minimally, a list of accessions and a genotyping protocol should be supplied. The required configuration fields for instantiation are ‘bcs_schema’ and ‘cache_root’ for the Bio::Chado::Schema database schema connection and the directory of the cache file system, respectively; all other fields are query parameters.

For convenience and performance reasons, the CXGN::Genotype::Search object provides three entry-points for retrieving results from the file cache, formatted as either VCF, dosage matrix, or internal JSON. There are two additional entry-points for retrieving genotypes in VCF and dosage matrix formats computed from genotyped parents; the progeny’s genotypes are calculated for each marker as an average of the parental dosage genotypes, simulating the inbreeding coefficient of each marker genotype of the hybrid as one-half of each of the two parents [22]. An example instantiation signature is given below with entry-points for retrieving each of the cache file formats. These entry-points have the advantage of being memory efficient by allowing reading of results line-by-line from the result file. The returned file handles can also be returned directly to the user for download, as is used for the Breedbase Search Wizard demonstrated in the “Web Interface Queries'' section below.

my $genotypes_search = CXGN::Genotype::Search->new({
 bcs_schema = >$schema,
 cache_root = >$cache_file_directory,
 accession_list = >\@accession_list,
 tissue_sample_list = >\@tissue_sample_list,
 trial_list = >\@trial_list,
 protocol_id_list = >\@protocol_id_list,
 markerprofile_id_list = >\@markerprofile_id_list,
 genotype_data_project_list = >\@genotype_data_project_list,
 chromosome_list = >[‘S1’,’S10’,’S80’],
 start_position = >‘9000’,
 end_position = >‘3000000’,
 marker_name_list = >['S80_265728', 'S80_265723'],
 genotypeprop_hash_select = >['DS', 'GT', 'DP'],
 protocolprop_top_key_select = >['reference_genome_name', 'header_information_lines', 'marker_names', 'markers'],
 protocolprop_marker_hash_select = >['name', 'chrom', 'pos', 'alt', 'ref'],
 return_only_first_genotypeprop_for_stock = >0,
 limit = >$limit,
 offset = >$offset
});
my @required_config = ($shared_cluster_file_directory, ‘Slurm’, ‘localhost’, ‘batch’, $basepath_directory);
# Retrieving VCF using cache file system
my $result_filehandle_VCF = $genotypes_search->get_cached_file_VCF(@required_config);
# Retrieving Dosage Matrix using cache file system
my $result_filehandle_dosage_matrix = $genotypes_search->get_cached_file_dosage_matrix(@required_config);
# Retrieving Internal JSON using cache file system. There is an option to retrieve metadata only without the genotype scores
my $result_filehandle_markerprofile_JSON = $genotypes_search->get_cached_file_search_json($shared_cluster_file_directory, $metadata_only);
# Retrieving VCF using cache file system for genotypes computed from genotyped parents
my $result_filehandle_VCF = $genotypes_search->get_cached_file_VCF_compute_from_parents(@required_config);
# Retrieving Dosage Matrix using cache file system for genotypes computed from genotyped parents
my $result_filehandle_dosage_matrix = $genotypes_search->get_cached_file_dosage_matrix_compute_from_parents(@required_config);

#### B. CXGN::Genotype::GRM

A Perl Moose object named CXGN::Genotype::GRM provides a standardized interface for retrieving a genomic relationship matrix (GRM) by minimally specifying a list of accessions and a genotyping protocol. The required configuration fields are ‘bcs_schema’, ‘people_schema’, ‘cache_root’, and ‘grm_temp_file’ for the Bio::Chado::Schema database schema connection, the CXGN::Metadata::Schema database schema connection, the directory of the cache file system, and a temporary file to save the GRM result, respectively; all other fields are query parameters and parameters for calculating the GRM. The GRM is computed using the R rrBLUP package and imputes missing genotypes using an ‘Expectation Maximization’ (EM) algorithm [23]. The genotypes are filtered using user input for minor allele frequency (MAF) and percent missing data for markers and samples prior to calculating the GRM. Three formats are available for download: a tab-separated matrix format (.tsv), a three-column format (.tsv), and a heatmap figure (.pdf). The three-column format is particularly useful for fitting mixed models in ASReml [24] once the data is exported from Breedbase. The GRM can be computed for accessions whose parents are genotyped, as described previously, using the ‘get_grm_for_parental_accessions’ boolean attribute.

my $geno = CXGN::Genotype::GRM->new({
 bcs_schema = >$schema,
 people_schema = >$people_schema,
 cache_root = >$cache_root,
 grm_temp_file = >$file_temp_path,
 accession_id_list = >\@accession_list,
 plot_id_list = >\@plot_id_list,
 protocol_id = >$protocol_id,
 get_grm_for_parental_accessions = >$compute_from_parents,
 download_format = >$download_format, #either ‘matrix’, ‘three_column’, or ‘heatmap’
 minor_allele_frequency = >0.01,
 marker_filter = >0.6,
 individuals_filter = >0.8
});
My $result_filehandle_grm = $geno->download_grm(@required_config);

#### C. CXGN::Genotype::GWAS

A genome-wide association study (GWAS) can be computed using the CXGN::Genotype::GWAS Perl Moose object by minimally specifying a list of accessions, a list of phenotypic traits, and a genotyping protocol. The required configuration parameters are ‘bcs_schema’, ‘people_schema’, ‘cache_root’, ‘grm_temp_file’, ‘gwas_temp_file’, and ‘pheno_temp_file’ for the Bio::Chado::Schema database schema connection, the CXGN::Metadata::Schema database schema connection, the directory of the cache file system, and temporary files to process the GWAS result, respectively; all other fields are query parameters and parameters for performing the GWAS. The R rrBLUP package is used to perform imputation using the ‘EM’ algorithm [23]; rrBLUP performs the GWAS using a mixed linear model including fixed effects for the experimental design (i.e., location and year of field experiment and replicate of tested accession) of the phenotypic measurements and using a kinship matrix calculated from the genotypic data. Genotypes are filtered by MAF and missing marker and sample data prior to calculating the kinship matrix and the GWAS. The GWAS can be computed for accessions whose parents are genotyped, as described previously, using the ‘get_grm_for_parental_accessions’ boolean attribute. If the provided trait list represents a series of repeated measurements, the boolean ‘traits_are_repeated_measurements’ attribute can be used. When traits are not to be treated as repeated measurements, results are returned for each trait independently. Results can be returned in two formats: a tabular result file (.tsv) or figures for Manhattan and QQ plots (.pdf).

my $geno = CXGN::Genotype::GWAS->new({
 bcs_schema = >$schema,
 people_schema = >$people_schema,
 grm_temp_file = >$file_temp_path,
 gwas_temp_file = >$file_temp_path_gwas,
 pheno_temp_file = >$file_temp_path_pheno,
 cache_root = >$cache_root,
 download_format = >$download_format, #either ‘results_tsv’ or ‘manhattan_qq_plots’
 accession_id_list = >\@accession_list,
 trait_id_list = >\@trait_id_list,
 traits_are_repeated_measurements = >$traits_are_repeated_measurements,
 protocol_id = >$protocol_id,
 get_grm_for_parental_accessions = >$compute_from_parents,
 minor_allele_frequency = >0.01,
 marker_filter = >0.6,
 individuals_filter = >0.8
});
My $result_filehandle_gwas = $geno->download_gwas(@required_config);

### III. Web interface queries

**Fig 2:**
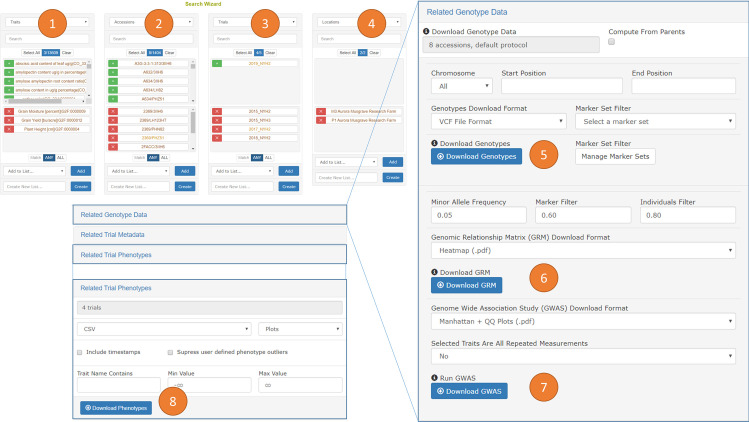
The search wizard is the primary means of querying Breedbase and provides a means for downloading phenotypic and genotypic records in several formats. The search consists of four query categories (1) to (4) to filter across every kind of data object in the database. In this example, traits were first selected (1) and ‘grain moisture’, ‘grain yield’, and ‘plant height’ were chosen. Then, accessions were selected (2) and from the 1,404 accessions which met the selected trait criteria 8 accessions were chosen. Then, trials were selected (3) and of the 5 field trials which met the selected trait and accessions criteria, 4 trials were chosen. Then, locations were selected (4) and the two locations which met the previous criteria were chosen. A genotyping protocol can be selected as a filter in (1) to (4); however, a default genotyping protocol is used when one is not explicitly selected. Clicking on “Related Genotype Data” brings a dialog to filter genotype data for the selected accessions by chromosome, start position, and end position prior to downloading in VCF or Dosage Matrix formats (5). Additionally, a marker set can be selected to filter downloaded genotypes further. Genotypes can be computed from parents in the pedigrees of the selected accessions if the parents were genotyped by clicking the “Compute from Parents” checkbox for (5), (6), or (7). The genomic relationship matrix (GRM) can be downloaded (6) for the selected accessions after filtering for minor allele frequency (MAF) and missing data. Three formats for downloading the GRM are available: a tab separated matrix format (.tsv), a three-column format (.tsv), and a heatmap figure (.pdf). A GWAS can be computed by selecting accessions and traits in (1) to (4) and results can be downloaded (7) as Manhattan and QQ plot figures (.pdf) or as a tabular file of the p-values (.tsv). Clicking “Related Trial Phenotypes” brings a dialog to filter phenotypes by minimum and maximum values prior to downloading phenotypic data in CSV or Excel formats (8).

Breedbase provides a web-interface compatible with all modern internet browsers on any device. A suite of web-pages are available for management of germplasm resources, pedigrees, seed inventories, field trials, experimental locations, phenotypic records, crossing blocks, genotyping storage, and other plant breeding program aspects. Once the information is entered into Breedbase, the primary means of searching and retrieving information is through the Search Wizard (Fig 2).

The Search Wizard (https://breedbase.org/breeders/search) enables construction of queries spanning accessions, field trials, genotyping protocols, locations, years, and phenotypic traits, and also provides an interface for downloading phenotypic and genotypic results as data files in several formats. Genotypic data can be filtered by chromosome, start position, and end position and can be downloaded in VCF or dosage matrix formats. More precise filtering is possible by selecting a marker set; a marker set is a user defined list of markers or a range of physical positions. Once accessions are selected the GRM can be downloaded, and if phenotypic traits are selected then a GWAS can also be downloaded. The Search Wizard internally uses the entry-points previously described in the “Packaged Queries” section. Phenotypic records can be filtered by minimum and maximum values prior to downloading as CSV or Excel files.

### IV. Limitations

The maximum number of markers that can be stored is limited by the PostgreSQL maximum data limit of 1GB per a single field [25]. If each marker requires 60 bytes of storage space, the maximum number of markers that can be described in a single genotyping protocol is approximately 17 million. For a 1 gigabase genome, this would represent almost 2 single nucleotide polymorphisms (SNPs) for every 100 base pairs or a polymorphism rate of about 2%. In the case of large genomes that are very polymorphic, these storage limitations may be reached, and additional implementation approaches may be required, such as storing different chromosomes individually in separate *genotypeprop* entries. Furthermore, storage of polyploid genotypes increases the number of bytes per marker, reducing the maximum number of markers which can be stored in the current implementation.

### V. Performance benchmark

To test the Breedbase JSON genotype storage system, three datasets were loaded into a test Breedbase instance running on a HP Z820 workstation with 256 GB RAM and 2x Intel Xeon E5-2660v2 CPUs. The first dataset loaded is a VCF containing 314 *Musa acuminata* samples genotyped with 142,119 genotype-by-sequencing (GBS) markers; this data is available in the Breedbase instance https://musabase.org/breeders_toolbox/protocol/1 and contains triploid genotypes [26, 27]. The second dataset loaded is a VCF containing 924 *Manihot esculenta* samples genotyped with 287,952 GBS markers and is available in the Breedbase instance https://cassavabase.org/breeders_toolbox/protocol/6 [28]. The third dataset loaded is a VCF containing 1,325 *Zea mays* samples genotyped with 955,690 GBS markers and is available in the Breedbase instance https://imagebreed.org/breeders_toolbox/protocol/5 [29].

Additionally, four phenotypic datasets were loaded into the Breedbase instance; the phenotypic records include accessions evaluated in field trials for which genotypic records in the aforementioned VCF datasets exist. The first dataset is from https://musabase.org of 75 phenotypic traits evaluated across 3 field trial experiments of *Musa acuminata* accessions. The second dataset is from https://cassavabase.org of 18 phenotypic traits evaluated across 3 field trial experiments of *Manihot esculenta* accessions. The third dataset is from https://imagebreed.org of 14 phenotypic traits evaluated across 3 field trial experiments of *Zea mays* accessions. To test computing genotypes from genotyped parents, pedigrees between hybrid *Zea mays* progeny accessions and parent accessions are uploaded into Breedbase; a fourth dataset was uploaded of 14 phenotypic traits evaluated across 3 field trial experiments for the hybrid *Zea mays* accessions.

The Perl test script, the three genotypic data VCF files, the four phenotypic data CSV files, and an SQL dump of the data loaded into the test Breedbase instance are included with this publication in the “Supplemental Information”. The phenotypic data files include the field experiment metadata and pedigree information. Note that there exist significant typographical errors between the accession names listed in the genotype VCF files and the accessions listed in the phenotypic information files, both for the tested accessions and for the pedigree accessions; however, Breedbase consolidates these names through a curation interface during upload of new accession names. Typographical errors such as ‘Tx303’ vs ‘TX-303’ are flagged by a text similarity score and the interface allows for correctly storing the relationships between identifiers.

#### Loading genotype data

For the benchmark test, all VCF files were uploaded consecutively through the Breedbase web-interface. Uploading the VCF containing 314 *Musa acuminata* samples genotyped with 142,119 GBS markers required a maximum of 2.36 GB RAM and 93 minutes to complete. Uploading the VCF containing 924 *Manihot esculenta* samples genotyped with 287,952 GBS markers required a maximum of 10.8 GB RAM and 237 minutes to complete. Uploading the VCF containing 1,325 *Zea mays* samples genotyped with 955,690 GBS markers required a maximum of 8.49 GB RAM and 535 minutes to complete. Future development to improve the upload process can parallelize genotype loading and can provide email responses to the user.

#### Retrieving genotype and phenotype data performance

For each of the three species in the test data, a random set of 25 accessions were chosen 10 different times with replacement. Those accessions were then (1) queried for 500 random markers and genotypic data was returned in VCF and dosage matrix formats, (2) the GRM was computed using all genotypes in the genotyping protocol after filtering for 1% MAF, 60% missing marker genotypes, and 80% missing sample genotypes, (3) GWAS was performed for two phenotypic traits using all genotypes in the genotyping protocol after filtering for 1% MAF, 60% missing marker genotypes, and 80% missing sample genotypes, and (4) the accessions were queried for all phenotypic traits evaluated. An additional scenario was tested for *Zea mays* in which the genotypes were computed from genotyped parents in the pedigree. Table 3 lists the mean query time in seconds for these four scenarios. The same queries were then performed a second time in order to test the file cache performance; Table 4 lists the mean query time in seconds for the four scenarios retrieving data from the file cache. The genotype downloads and computation of the GRM and GWAS were performed using the CXGN::Genotype::Search, CXGN::Genotype::GRM, and CXGN::Genotype::GWAS modules described in the “Packaged Queries” section.

**Table 3:** Results for non-cached query performance.

Crop Species Test Scenario For Non-cached Query	VCF Mean Download Time (s)	Dosage Matrix Mean Download Time (s)	Genomic Relationship Matrix (GRM) Mean Download Time (s)	Genome Wide Association Study (GWAS) Mean Download Time (s)	Phenotype Query Mean Time (s)
*Musa acuminata* (triploid accessions)	129.8	99.7	257.3	373.7	0.220
*Manihot esculenta* (diploid accessions)	240.8	159.3	649.7	810.3	0.030
*Zea mays* (diploid hybrid genotypes calculated from genotyped parents)	1083.7	874.7	6696.6	7570.1	0.040
*Zea mays* (diploid accessions)	454.6	634.5	5973.5	6347.2	0.040

Mean time in seconds required to download VCF, dosage matrix, GRM, GWAS, and phenotypic results for the banana, cassava, and maize test datasets loaded into a test Breedbase instance. The maize data tested an additional scenario in which the hybrid genotypes are computed from their genotyped parents in the pedigree.

**Table 4:** Results for repeated query performance from the file cache.

Crop Species Test Scenario For Repeated Cached Query	VCF Mean Download Time (s)	Dosage Matrix Mean Download Time (s)	Genomic Relationship Matrix Download Time (s)	Genome Wide Association Study (GWAS) Mean Download Time (s)	Phenotype Query Mean Time (s)
*Musa acuminata* (triploid accessions)	0.010	0.010	0.007	0.007	0.220
*Manihot esculenta* (diploid accessions)	0.009	0.010	0.007	0.008	0.030
*Zea mays* (diploid hybrid genotypes calculated from genotyped parents)	0.007	0.018	0.007	0.007	0.040
*Zea mays* (diploid accessions)	0.010	0.029	0.008	0.007	0.070

Mean time in seconds required to download VCF, dosage matrix, GRM, GWAS, and phenotypic results for the banana, cassava, and maize test datasets loaded into a test Breedbase instance. The maize data tested an additional scenario in which the hybrid genotypes are computed from their genotyped parents in the pedigree.

### VI. Scalability and continued development

Cassavabase (https://cassavabase.org) is the Breedbase instance currently with the largest genotypic data, nearly 100,000 samples with dense GBS genotypes from genotyping protocols of up to 287,952 markers, as well as thousands of samples with low density genotypes from genotyping protocols of around 20 markers. The system is used routinely to perform genomic selection analysis by affiliated breeding programs using the built-in solGS tool [6]. Further scalability tests and development will be necessary before this solution can accommodate very large breeding programs; however, Cassavabase and the performance benchmark described above show that for small to medium programs the implementation presented here is an appropriate solution.

Development will continue on the presented database system to support the many plant breeding communities relying on Breedbase. Software development of Breedbase is streamlined through the Git version control available on Github through https://github.com/solgenomics/sgn. In this way, issues arising in the software can be posted and new releases to the software can be managed through a review process. Documentation is bundled directly with the software at https://solgenomics.github.io/sgn. For easy deployment, Breedbase is released in a Docker https://github.com/solgenomics/breedbase_dockerfile. The Docker deployment allows launching a Breedbase web-server and database instance with minimal configuration, and provides detailed documentation.

## Supporting information

S1 Appendix (TXT)

S1 File (PL)

S2 File (CSV)

S3 File (CSV)

S4 File (CSV)

S5 File (CSV)
